# 

**Published:** 1882-08

**Authors:** 


### CONTENTS.

### *MISCELLANEOUS.*

| | PAGE. |
|---|---|
| Homœopathy or Eclecticism? Editorial, | 321 |
| The Duties of the Hour. C. Pearson, M. D., | 323 |
| Materia Medica and Its Practical Uses. P. P. Wells, M. D., | 335 |
| Clinical Reflections. Ad. Lippe, M. D., | 347 |

### *CLINICAL BUREAU.*

| | |
|---|---|
| Fistula Lachrymalis and Mylopetroleum Ointment. C. F. Nichols, M. D., | 349 |
| A Contribution to Surgical Therapeutics. John Hall, M. D., | 350 |
| Senile Gangrene: Amputation of Thigh. J. B. Bell, M. D., | 353 |
| Surgical Therapeutics. J. R. Haynes, M. D., | 357 |

### *BOOK REVIEWS.*

| | |
|---|---|
| Diet of Infants and Young Children. By J. C. Morgan, M. D., | 359 |
| Eaton's Domestic Practice for Parents and Nurses. By Morton M. Eaton, M. D., | 359 |
| The Address before the American Institute. By the President, Dr. Breyfogle, | 359 |
| The Diseases of the Pancreas, and their Homœopathic Treatment. By Drs. Farrington, Karndœrfer, Morgan and Thomas, | 360 |
| Errata, | 360 |
| Cough Symptoms, | 17–24 |

